# Ligands stimulating antitumour immunity as the next G-quadruplex challenge

**DOI:** 10.1186/s12943-022-01649-y

**Published:** 2022-09-17

**Authors:** Giulia Miglietta, Jessica Marinello, Marco Russo, Giovanni Capranico

**Affiliations:** Department of Pharmacy and Biotechnology, Alma Mater Studiorum University of Bologna, via Selmi 3, 40126 Bologna, Italy

**Keywords:** G-quadruplex, G4 ligands, G-loop, Genome instability, Innate immunity, Cancer immunotherapy

## Abstract

G-quadruplex (G4) binders have been investigated to discover new anticancer drugs worldwide in past decades. As these ligands are generally not highly cytotoxic, the discovery rational was mainly based on increasing the cell-killing potency. Nevertheless, no G4 binder has been shown yet to be effective in cancer patients. Here, G4 binder activity at low dosages will be discussed as a critical feature to discover ligands with therapeutic effects in cancer patients. Specific effects of G4 binders al low doses have been reported to occur in cancer and normal cells. Among them, genome instability and the stimulation of cytoplasmic processes related to autophagy and innate immune response open to the use of G4 binders as immune-stimulating agents. Thus, we propose a new rational of drug discovery, which is not based on cytotoxic potency but rather on immune gene activation at non-cytotoxic dosage.

## Background

**Fig. 1:**
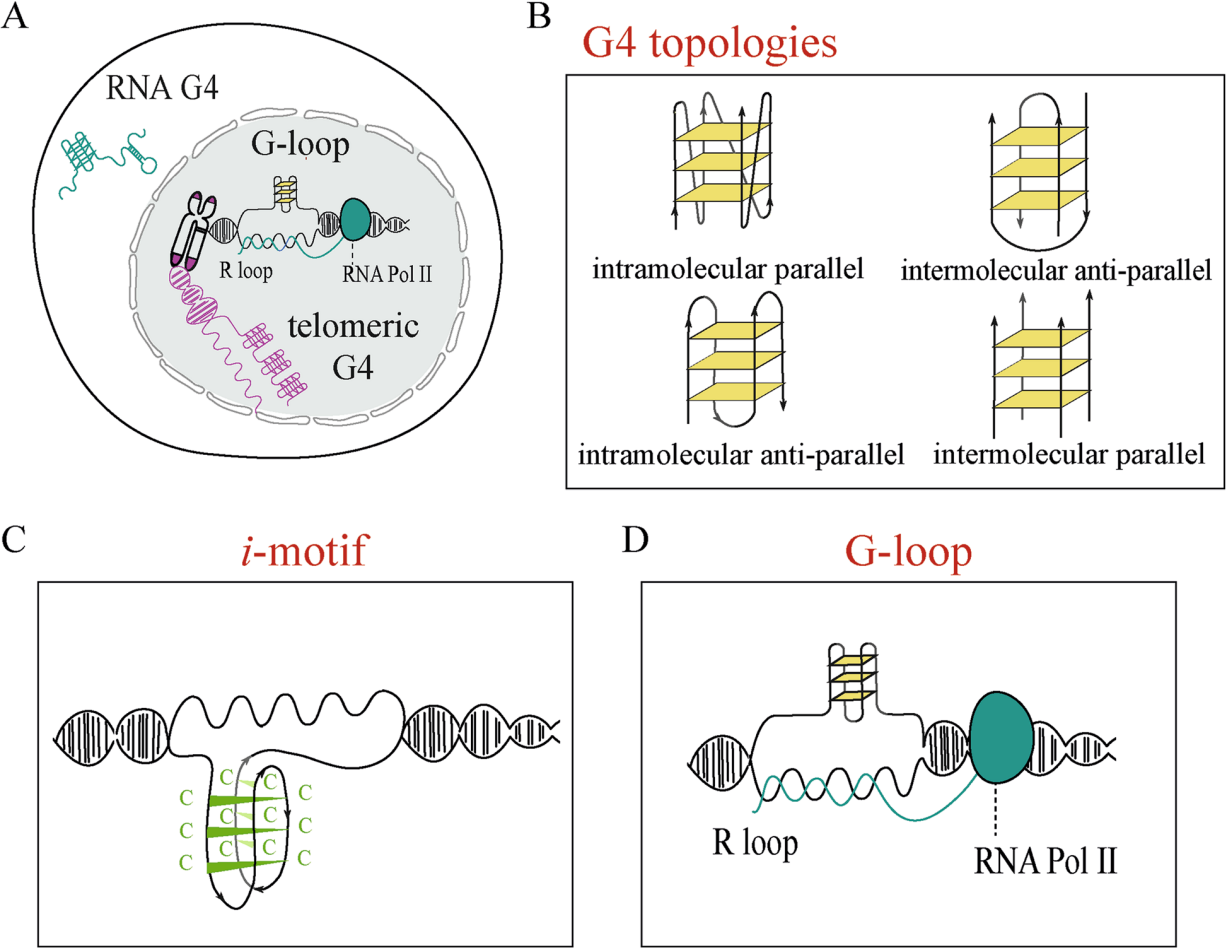
G-quadruplex and other non-B DNA structures. G-quadruplexes can form in G-rich strand of both RNA and DNA strands (**A**). They can fold into different conformations depending on strand stoichiometry (intra- or inter-strand topologies), strand direction (parallel, antiparallel, or mixed) and length of intervening nucleotides (bulges and loops) (**B**). C-rich DNA strands can fold into *i*-motif structures (**C**). R-loops can favour G4 folding on the displaced DNA strand resulting into another non-B structure known as G-loop (**D**)

Even though genomic DNA is generally arranged into the canonical Watson-Crick B-form duplex, structural conformations can vary as DNA and RNA can form several non-B secondary structures in living cells, including G-quadruplex (G4) (Fig. 1A). G4 is constituted by a pile of two or more planar G-tetrads, formed by four guanines held together by Hoogsteen hydrogen bonds. The stacking of G-tetrads is stabilized by K^+^ or Na^+^ located at the tetrad centre. Experimental and bioinformatic data showed that the canonical consensus of potential G4-forming sequences (PQS) is G_≥3_N_1–7_G_≥3_N_1–7_G_≥3_N_1–7_G_≥3_ [1], even though a majority of sequences shown to form a G4 structure can escape the consensus [2]. The ability of a G-rich strand to adopt a G4 secondary structure was demonstrated in 1962 [3] and, after decades of studies, we now know that runs of DNA and RNA guanines can adopt very polymorphic G4 structures. A G-rich strand can fold into different G4 conformations depending on strand stoichiometry (intra- or inter-strand topologies), strand direction (parallel, antiparallel or mixed) and length of intervening nucleotides (loops and bulges) [4, 5] (Fig. 1B).

G4 folding in vivo has been visualized by using specific antibodies (BG4) in fixed cells [6] or fluorescent and radioactive probes in living cells [7–10]. Several genetic data further demonstrate the occurrence of G4 structures in the genome of humans and several other organisms, including bacteria and viruses [11, 12]. According to computational tools and genomic G4 mapping, G4s can occur across the genome, particularly enriched in gene regulatory regions and telomeres supporting fundamental functions [13, 14]. For instances, G4s have been shown to exert beneficial effects on homeostatic molecular processes such as telomere maintenance, immunoglobulin recombination, and regulation of gene transcription and mRNA translation [9, 15].

**Fig. 2:**
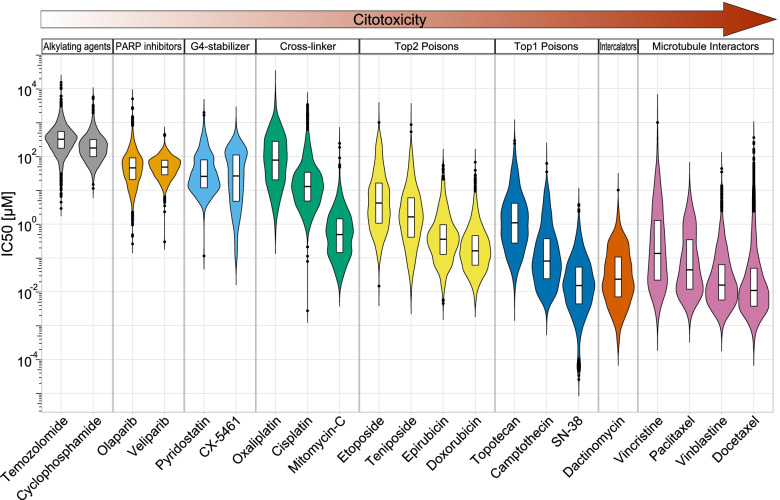
Cytotoxicity potency of anticancer  DNA-interacting agents. Violin plots showing IC_50_ values of selected DNA-interacting agents with established anticancer activity in standard chemotherapy of human tumours. Data were obtained from “Genomics of Drug Sensitivity in Cancer” (GDSC) database [40], which includes information from around one thousands cancer cell lines (www.cancerrxgene.org). Drugs are classified on the base of their mechanism of action and ranked by cytotoxicity (left to right). G4 stabilizers have a lower cytotoxic potency than topoisomerase poisons, microtubule interactors, Mitomycin C and Dactinomycin (Actinomycin D). Alkylating agents and cross-linkers can react with cytoplasmic nucleophilic groups in cells, effectively lowering their nuclear concentrations [41]. Moreover, PARP inhibitors are mainly used in BRCAness tumours where their cell killing potency is much increased due to mutations or deletion of BRCA genes [42]. Therefore, G4 binders (Pyridostatin and CX-5461) have a low cytotoxic potency in comparison to other effective DNA-interacting anticancer agents

In the past, G4s in telomeres and oncogene promoters have been investigated as targets of specific ligands (G4 binders) to discover chemotherapeutic agents for the treatment of cancers addicted to oncogenes and/or sensitive to telomere loss [16–18]. More recently, several structurally-diverse G4 binders were shown to inhibit and arrest DNA replication leading to DNA damage and genome instability [19–22]. In addition, as nuclear levels of G4 structures can be higher in cancer than normal cells [14, 23, 24], published data converge on G4s as potentially-effective pharmacological targets for cancer treatment. Therefore, past research in the field focused on developing more cytotoxic ligands, some of which have shown antitumour activity in human tumour xenograft models, in particular in combination regimen or in homologous recombination (HR) repair-deficient tumours [25, 26]. However, despite decades of research in the field, only two G4 binders (CX-3543 and CX-5461) reached early phases of clinical trials (Tables 1 and 2), and none has been shown definitively to possess effective anticancer activity in patients. The cell-killing potency of G4 binders are significantly lower than DNA-interacting agents with established antitumour activity in patients, such as topoisomerase poisons or microtubule-interacting agents (Fig. 2). A low cell-killing potency can be a peculiar feature of G4 binders which can be difficult to overcome to achieve an effective antitumour activity in patients. Interestingly, results of a Phase I trial of CX-5461 have published very recently showing a 14% partial responses in female patients with cancers with mutations of BRCA1/2 or PALB2 genes [27]. Reversion mutations predicted to restore HR capability were associated with disease progression [27], suggesting a link between the compound efficacy and HR deficiency, which may eventually be used to select patients for CX-5461 treatment. Thus, similarly to PARP inhibitors (Fig. 2), a higher efficacy in those patients is consistent with a higher cytotoxic potency of CX-5461 in HR-defective cancer cells [26].

**Table 1:** Clinical studies of G-quadruplex binders

Trial identifier	Compound	Clinical phase	Cancer type	Status/Publication	Country	Year ^a^
NCT00955786	CX-3543 (Quarfloxin)	Phase 1	Advanced solid tumour Lymphoma	Completed	USA	2009
NCT00955292	CX-3543 (Quarfloxin)	Phase 1 (modified schedule)	Advanced solid tumour Lymphoma	Terminated	USA	2009
NCT02719977	CX-5461 (Pidnarulex)	Phase 1	Cancer	Active, not recruiting / [27]	Canada	2016
NCT04890613 ^b^	CX-5461 (Pidnarulex)	Phase 1b	Advanced solid tumour	Recruiting	Canada	2021

The information can be found at Clinicaltrials.gov and in the indicated publications.

^a^Year indicates when the study has been started.

^b^This trial aims at defining predictive values of mutational BRCA2 and PALB2 gene signatures along with tolerable CX-5461 doses for phase II studies.

**Table 2:** G4-quadruplexes binders and their known effects

Compound	Structure	G4-dependent effects	Ref.
20A		• Autophagy • Cell senescence	[28]
Ant 1,5		• Autophagy	[29]
AQ1		• Oncogene targeting • Oncogenic cell death • Cell survival inhibition	[30, 31]
Braco-19		• Telomere targeting • Transcriptional landscape alteration	[32]
CM03		• Oncogene targeting • Oncogenic cell death • Cell survival inhibition	[30, 31]
CX-3543 (Quarfloxin)		• Antitumor activity in mice	[33]
CX-5461 (Pidnarulex)		• Higher cytotoxic potency in BRCA2-mutated cancer cells • Partial responses in cancer patients with BRCA1/2 or PALB2 mutations	[25–27]
Emetine		• Transcriptome-wide downregulation	[34]
FG		• R-loop formation • Micronuclei formation • DNA damage	[20]
Pyridostatin		• DNA damage • R-loop formation • Innate immune genes activation • Micronuclei formation	[19, 20, 35]
PhenDC3		• Micronuclei formation • Innate immune gene activation • Epigenetic regulation of gene expression	[35, 36]
Pt1		• Telomerase inhibition • Cancer cells apoptosis	[37]
RHPS4		• Telomere targeting • Apoptosis • Transcriptional landscape alterations • B-cell proliferation arrest	[32, 38]
SYUIQ-5		• Telomeric DNA damage	[39]

At the same time, the mechanistic understanding of G4 action in cancer cells may open to unpredicted strategies, unrelated to cytotoxicity, to discover effective anticancer G4 binders. Recent advances have clearly established a complex crosstalk between genome instability and the immune system as cytoplasmic DNA/RNA can function as an alarm molecule that activates innate immune gene pathways in cancer cells. These cellular processes have the potential of harnessing the innate immune system for cancer immunotherapy [43]. Thus, in this review, we discuss published data on the mechanisms through which G4 stabilization can trigger genome instability eventually leading to immune gene activation and related processes in cancer vs normal cells. Firstly, we discuss mechanisms of DNA damage and replication stress leading to genome instability. Then, we discuss the induction of cytoplasmic processes signalling an alarm for the cell and leading to immune gene response and/or autophagic processes, in relation to therapeutic activity of G4 binders and established anticancer drugs. G4-mediated genome instability and activation of specific cytoplasmic pathways constitute a key point to open promising lines of drug discovery as the next challenge in the field is the development of G4 binders as potent stimulators of antitumour immunity at non-cytotoxic dosage.

## Main text

### G-quadruplex structure, such a desirable target

G4 structure has been and still is an attractive target for specific ligands with antitumour activity. The diversity of G4 structural features, such as G-tetrad length and loops, can offer an effective way to design G4-selective small molecules. Historically, G4 binders were designed to target telomeres with the aim to inhibit telomerase activity and consequently proliferation rate of immortalized cancer cells [44, 45]. Successively, the observation that G4s are enriched in many genomic regulatory regions, oncogene promoters and open chromatin regions, revealed new opportunities for ligands to selectively target G4s regulating oncogene expression [46]. Many efforts have been focused on trying to inhibit single oncogene expression to exploit the *tumour addiction* phenomena [47] and enhance G4-binder cancer cell killing potency. This strategy may be a solution for undruggable proteins, such as RAS in *RAS*-driven tumours [16].

Nevertheless, small molecules with high G4 affinity likely target multiple G4s in the genome leading to a wide and complex alteration of cellular transcriptomes. The diversity of this class of molecules is high (> 3000 compounds) [48], including also metal-containing compounds (as the organoplatinum complex Pt1, Table 2) with cell killing potential and ability to induce cell senescence [37, 49]. However, studies focusing on G4-binder genome-wide effects are still a low number. RNA seq experiments revealed that the G4 binders, CM03 and AQ1 (Table 2), can globally alter gene expression and inhibit pathways involved in cell survival [30], tumour progression and oncogene Kit- and Myc-related processes [31]. Braco-19 and RHPS4 (two G4 binders originally developed to target telomeres, Table 2) can alter the full transcription landscape supporting pleiotropic effects well beyond telomere stability [32]. Gene expression profiles were shown to be specifically altered by Pyridostatin (PDS, Table 2) at later times from treatment in human cancer cells [35]. In this study, PDS was used at non cytotoxic concentrations and the findings reveal a specific enhancement of expression of innate immunity-related genes [35]. Moreover, gene expression can be modulated by interfering with RNA G4s and thus by altering the transcript stability, mRNA splicing and translation. Interestingly, eukaryotic initiation factor 4A (eIF4A) inhibition, promoted by emetine (Table 2), caused the translational downregulation of many genes with potential quadruplex-forming sequences in their 5′-UTRs [34]. Thus, published findings on transcriptome alterations by G4 binders show that they exert a broad effect on gene expression, suggesting that single-gene expression alterations are unlikely to be achieved and any biological or therapeutic activity would rather be a consequence of an overall transcriptional change. Moreover, mechanisms through which G4 binders affect gene transcription are still not fully understood. A recent study shows that a G4 motif positioned downstream of the transcription start site (TSS) can influence transcription efficiency differently, depending on its orientation [50]. In particular, a G4 in the template strand inhibits RNA polymerase elongation, whereas a G4 in the non-template strand affects transcription depending on the specific genomic and chromatin context [50].

The published findings on transcriptional effects of G4 binders have been obtained at different times, in different cell lines and with different concentrations, and many of them were designed to get insights into cell killing mechanisms. However, the great diversity of experimental conditions cannot allow to derive a general conclusion on specific pathways altered by G4 binders and further mechanistic insights are needed to exploit transcription effects of G4 binders for therapeutic purposes.

### G-quadruplex interactions with other non-canonical DNA/RNA structures

Since the first demonstration of a G4 structure in vitro [3], research has been focused at the demonstration that G4s exist in living cells in both the genome and transcriptome [51]. Several experimental data now support not only the occurrence of RNA and DNA G4s in vivo, but also their involvement in the regulation of physiological and pathological processes [9]. G4s are highly dynamic structures and their steady-state levels are generally low in nuclei of unperturbed living cells [52]. As G4s can form in single-stranded DNAs, they cannot easily fold in the context of genomic DNA as strand pairing of the DNA duplex strongly opposes the formation of alternative secondary structures of single DNA strands. Consistently, G4 folding is thermodynamically favoured when strand separation has occurred, such as during replication or transcription [22].

When DNA is not replicating, the transition of DNA from canonical B-form to G4s or alternative secondary structures likely requires strand unwinding of the double helix by local negative DNA supercoils [52, 53]. Hurley and co-workers clearly established the importance of negative super-helicity for non-B structure folding at the c-MYC promoter [54, 55]. As transcription elongation is the main source of torsional stress and unwinding of the genome, G4 folding at transcribed genes can then be regulated by the stabilization of the opposite DNA strand by either protein factors or other secondary structures [56]. The formation of *i*-motifs (Fig. 1**C**), through hemi-protonated cytosine-cytosine base pairing (C-C+) [57, 58], could stabilize the opposite C-rich DNA strand. However, single-molecule studies showed a mutual exclusivity between *i*-motifs and G4s at the Myc promoter [59] and other gene promoters [60, 61]. As simultaneous formation of the G4s and *i*-motifs in opposite strands occurs only when they are placed in an offset fashion [61], the mutual exclusivity is likely due to steric hindrance of the two structures. Thus, *i*-motifs cannot stabilize the opposite strand of a G4 structure. In addition, chemical G4 stabilization has been shown to destabilize *i*-motifs in cultured cells, and vice versa [62], suggesting an interplay of G4 and *i*-motif formation in relation to DNA function regulations in human cells [59, 62]. Further investigations should thus define whether G4 binders may achieve a biological activity by interfering indirectly with *i*-motif formation.

Structural features that favour G4 folding (GC-richness, strand separation, negative supercoils) also favour the formation of R-loops, another non-B structure [20, 24, 63]. R-loops, commonly occurring co-transcriptionally, are three-strand nucleic acid structures, wherein the nascent RNA strand is annealed to the template DNA strand forming a hybrid duplex, while the non-template DNA strand is displaced out. Thus, R-loops can favour G4 folding on the displaced DNA strand resulting into a new structure known as G-loop (Fig. 1D) [20, 50, 63, 64]. R-loop formation is a dynamic event at active genes, and they can cover 4–7% of a cell genome [65]. They are key players in many biological processes such as replication, transcription activation and termination, and are crucial for immunoglobulin class-switch recombination (CSR) in activated B lymphocytes [66]. G4 and R-loop co-existence has been disclosed in human cells by the overlapping of nuclear foci of these two non-B DNA structures visualized with specific antibodies (BG4 and S9.6, respectively) [20, 24, 50]. In particular, kinetics of G4 and R-loop formation by cell treatments with G4 binders are very similar in human U2OS cells [20]. PDS (Table 2) can modulate R-loop formation promoting G-loop structures by extending transcriptional DNA:RNA hybrids [20] (Fig. 1D). Interestingly, G4/R-loop interplay can be the key point to disclose the dynamics involved in genome instability caused by G4 structures.

### G4 stabilization, replication-dependent DNA damage and genome instability

G4 folding can represent a steric obstacle to the elongation of the replication machinery. Evidence proved that G4 structures can stop DNA polymerases and impair replication fork progression in vitro [67–69]*.* In particular, replication forks using a telomeric G-rich strand template proceed slower than adjacent non G-rich DNAs and fork progression is further slowed down in the presence of a G4 binder [70]. Therefore, prokaryotic and eukaryotic cells are equipped with a robust system of specialized helicases that effectively unfold G4s during DNA replication allowing an effective fork progression [70–72]. The activity of G4 helicases is a critical step to overcome G4 obstacles and minimize DNA damage during the replication process. The first evidence that G4 can compromise replication fork progression in vivo was reported in *C. elegans* as a *DOG1* (*FANCJ*) gene deletion could lead to accumulation of small deletions upstream to putative G4s [73]. Moreover, Pif1, an efficient G4 helicase, is fundamental to maintain genome stability in *S. cerevisiae* by preventing replication-dependent DNA damage at G4 motifs [74]. *PIF1* gene depletion is also responsible for genetic and epigenetic G4-mediated changes [75, 76]. In mammalian cells, BLM helicase activity has been shown to resolve G4s and disrupt steric impediments during replication preventing genome instability, as *BLM* gene depletion resulted in increased levels of stabilized G4s and telomere fragility in murine fibroblasts [70]. A coordinated action of three helicases, DHX35, FANCJ and the replicative CMG complex, has recently been shown to promote bypass and unfolding of stabilized G4s during replication in an ex-vivo model of *Xenopus* egg extracts [77]. Mammalian telomeres are also recognized by the ssDNA-binding complex CST (CTC1-STN1-TEN1), which can target and unwind G4s in vitro. In particular, STN1 localization is altered after treatment with different G4 binders suggesting that G4 stabilization triggers the CST complex redistribution at specific genome sites to reduce G4 accumulation and to allow replication process [78, 79].

Moreover, DNA synthesis re-priming can overcome a stable G4 through an obstacle-bypass mechanism. Sale and co-workers [80–82] showed that Primase-Polymerase (PrimPol) is critical for DNA damage tolerance, and binds to G4 structures while re-priming DNA leading strand synthesis downstream to G4s. Therefore, PrimPol activity allows the restart of fork progression bypassing the structural obstacle and prevents genomic instability [80]. Interestingly, when a stable G4 is not resolved by helicases at replication forks, it can persist through the next mitotic division along with a single-strand gap in nascent DNA, due to failed replication. The single-strand gap then becomes double-strand break (DSB) during S-phase of daughter cells, which can eventually repair DSB by alternative Polθ-end joining pathway [83], causing an increase of genome instability.

Unbalanced G4 levels promoted by some G4 binders can be responsible for DSB accumulation, cell-cycle arrest at G2/M phase and activation of DNA damage response (DDR) pathways [19, 20, 83–85] suggesting that DNA cleavage activity could be exploited to discover new G4 binders with anticancer activity. DSB may be formed through several mechanisms [22], however R-loops can mediate either replication- and transcription-dependent DSB generation from chemical stabilization of G4s [20, 22]. Recent studies also revealed that homologous recombination repair (HRR) plays a pivotal role in cell survival as demonstrated by the increased cytotoxicity of G4 binders in BRCA1/2-deficient cancer cells [25, 26]. HRR factors BRCA1 and BRCA2 have several specific functions and are recruited to prevent DNA degradation at stalled replication forks [86, 87], to promote the restart of replication and to drive HRR processes [88]. G4 stabilization promoted by specific ligands is clearly responsible for genome-wide DSB accumulation in cancer cells. However, it is worth noting that, on the one hand, unrelated G4 binders have a different propensity to induce DNA cleavage in cancer cells [22] and on the other, DSBs are induced by G4 binders at non-cytotoxic dosage [19, 20, 89]. Therefore, cancer cells can often survive while overcoming G4 binder-induced DSBs, which may be more related to cell senescence rather than cell death or apoptosis.

Interestingly, PDS and FG (Table 2) have been shown to promote R-loop-mediated micronuclei formation in human cancer cells and BRCA2 silencing increased micronuclei levels with respect to BRCA2 proficient cells [20]. Micronuclei are chromatin portions separated from the main nucleus and with their own membranes, which are generated at mitosis through aberrant chromosome segregation [90]. Recent findings showed that chemically-unrelated G4 binders are able to induce micronuclei accumulation in different cancer cell lines [35, 89], indicating that micronuclei induction is a common effect of many G4 binders. Micronuclei are readily induced by non-cytotoxic PDS concentrations in breast MCF-7 cancer cells [35] indicating that DSBs and genome instability are not associated to cytotoxicity. Moreover, the definition of transcriptome profiles established that the G4 binder induced innate immune genes through the activation of IRF1–7 transcription factors [35]. Innate immune genes were induced through the activation of cGAS-STING-IRF3 signalling pathway upon PDS-triggered micronuclei increase [35]. These findings open new frontiers in the G4 and oncology fields to discover new G4 binders as non-cytotoxic immunomodulatory agents to be used in combination with immunotherapies.

### G4 binders as modulators of innate immunity genes and autophagy in cancer cells

**Fig. 3:**
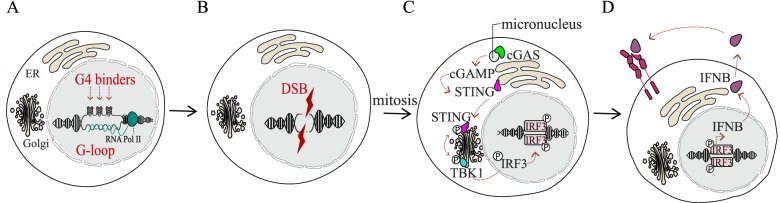
G4 binders increase R-loop-dependent genome instability activating then the cGAS/STING/IRF3 pathway in cancer cells. Mechanism of G4-binders activity in cancer cells. A) G4 binders target G4 structures by stabilizing the G-loop. B) G-loops can induce double strand breaks through either replication- and transcription-dependent mechanisms, resulting in cell senescence or micronuclei induction. C) Micronuclei are recognized by DNA sensing protein cGAS, leading to activation of cGAS/STING pathway and to IRF3- regulated gene expression. D) The phosphorylated form of IRF3 translocates to the nucleus and promotes IFNB expression in cancer cells. IFNB secretion allows its binding to a membrane interferon receptor and consequent IFNB-dependent transcriptional cascade activation.

The latest and successful progresses in medical oncology have been achieved by the development of effective anticancer immunotherapy [91, 92]. Immunotherapies aimed to enhance T-cell responses by targeting inhibitory pathways using immune checkpoint inhibitors [93] or by stimulation using chimeric antigen receptor T cells (CAR-T) or bispecific antibodies [94]. Unfortunately, many tumours do not respond to them or become unresponsive at relapse [95], highlighting the need to identify novel mechanisms and/or molecules able to optimize immunotherapeutic strategies. The T-cell activation depends on the innate immune response which is the first barrier of defence to monitor and detect molecular alterations in cancer cells [96]. Interestingly, the activity of G4 binders can activate a physiological process, known as Pathogen- or Damage-Associated Molecular Patterns (PAMPs or DAMPs), activated mainly in immune cells by microbial or viral infections through the activity of Pattern Recognition Receptors (PRR) [97]. In particular, two chemically-unrelated G4 binders (PDS and PhenDC3, Table 2) have been shown to activate an innate immune cascade in human breast cancer cells through micronuclei accumulation at non-cytotoxic concentrations [35]. DNA damage promoted PDS and PhenDC3 results into micronuclei formation in different cancer types such as human osteosarcoma, and murine melanoma cells [20, 35, 89]. Micronuclei are recognized by specific PRR or cytoplasmic DNA sensors like cGAS [98, 99], and cGAS bound to micronuclei leads to STING activation, which is a pivotal player in promoting Type I Interferon and other immune genes [100] as silencing, deletion or chemically inhibition of STING abolished immune genes response in cancer cells (Fig. 3). In our report, non-cytotoxic concentrations of G4 binders were used and the transcriptome changes were determined following 3 days of cell recovery in drug-free medium [35]. This has allowed the determination of transcription profile changes at later time from drug treatments, which are likely more lasting than changes at short times. Gene expression data showed that PDS stimulates a subset of immune-related pathways such as response to type I interferon, lymphocyte and T cell migration and autophagy-related pathways. The findings strongly support a novel biological outcome of G4 binders at non-cytotoxic doses that can be exploited to stimulate antitumour immunity [35] (Fig. 3). The STING-dependent type I interferon response plays a role in T-cell tumour recruitment fundamental to elicit the immune adaptive tumour surveillance, as STING activation could mediate the tumour response to the checkpoint inhibitors as shown by the reduced efficacy of the anti-CTLA4 and anti-PDL-1 treatment in STING-deficient mice [99, 101, 102]. The ability of STING to mediate the anticancer activity of checkpoint inhibitors highlights an attempt to exploit G4-mediated STING activation for treatments of unresponsive tumours. In this scenario, the action of non-cytotoxic G4 binder doses may play a further role to promote an immune-therapeutic activity as immunotherapy efficacy is strictly related to the mutational burden of tumours [103, 104]. Since the mutagenic burden of tumours correlates with the immunotherapeutic efficacy, G4 binder-mediated genome instability may even enhance tumour immunogenic responsiveness [105].

Microbial DNAs can act as a DAMPs in infected mammalian cells, in particular bacterial CpG-rich DNAs are most effective in the activation of this process. Interestingly, synthetic DNA oligomers bearing unmethylated CpG can trigger an immune response in a way consistent with bacterial DAMPs [106]. The presence of a G4 in the oligomer can increase the immunostimulation through activation of TLR9, which can bind and recognize G4 structures [107]. The addition of G4 binder can however have opposite effects, as the immunostimulatory features of an oligomer containing a single hybrid-type G4 depend on the capacity of tested ligands to stabilize and maintain the hybrid quadruplex topology [107]. Thus, it remains to be established whether abnormally-increased endogenous DNA/RNA G4s may directly bind to PRRs, such as TLR9 and likely others present in the cytoplasm or cellular membranes, to activate immune genes in cancer cells as well.

Along with innate immune gene activation, PDS can also trigger autophagic pathways in human MCF-7 breast cancer cells [35]. This is consistent with other findings showing that the cGAS-STING pathway activates the autophagy process through a TANK-binding kinase 1 (TBK1) independent mechanism [108] and that cGAS is a critical regulator of inflammation and autophagy in Huntington’s disease [109]. In addition, micronuclei levels are increased in cells derived from Aicardi-Goutières syndrome (AGS) patients, a disease characterized by an excess of interferon production and inflammation due to an overactivation of the cGAS/STING pathway [110]. The excess of micronuclei in AGS cells also stimulates autophagy, which in turn targets micronuclei for lysosomal degradation, therefore preventing further stimulation of the innate immune response [110]. Interestingly, an enhanced autophagy due to mTOR inhibition by rapamycin leads to micronuclei resolution [110].

Independent studies showed that G4 binders induce autophagy even though published reports do not agree on the biological outcomes, likely due to differences of experimental conditions or cancer cell type. The anthracene derivative Ant1,5 (Table 2) stimulates the autophagic marker LC3B inducing autophagosome accumulation in cells, suggesting autophagy as a defence against ligand-mediated DNA damage [29]. In contrast, SYUIQ-5 (Table 2) has been shown to promote telomeric DNA damage by TRF2 delocalization and an ATM-dependent autophagic cell death [39]. More recent transcriptomic analyses revealed that the G4 binder 20A (Table 2) promotes cellular pathways related to growth arrest and lysosomal signalling showing that ATM activation is functional for autophagy induction and cell senescence [28]. The study presents the ATM/autophagy axis as a key player for cell fate between senescence and apoptosis, therefore suggesting that autophagy can be a target to induce G4 ligand-dependent cancer cell death [28].

Altogether, the results show that chemically unrelated ligands promote autophagy in cancer cells, likely dependent on the activation of ATM and cGAS/STING pathways. Autophagy can then lead to cell death or survival depending on the magnitude and duration of the activation and the cell genetic background. Moreover, as autophagy is involved in micronuclei clearance [111], the functional effect of autophagy needs to be fully understood in relation to the potential role of G4 binders as immunomodulators of antitumour immunity.

### G4 binder activity in normal cells

G4 and R-loop structures have physiological roles in complex mechanisms relevant for cell life and organism development. Thus, the understanding of G4 binder effects in normal cells is essential to discover therapeutic activity of G4 binders for either cancer or other diseases. Interestingly, low doses (sub-cytotoxic) of RHPS4 (Table 2) directly target CSR of immunoglobulin genes in B cells, while neither decreasing B-cell growth nor triggering apoptosis. Ligand targeting CSR can lead to a decrease of pro-inflammatory class-switched immunoglobulins impacting on immune-allergic conditions in mice [38]. In particular, the authors demonstrate that, in stimulated primary mouse B-cells, a 10-fold reduction of secreted class-switched immunoglobulin occurs after drug treatment along with a reduction of Activation-Induced Deaminase (AID) recruitment at Sμ, Sε and Sγ1 regions [38]. Similarly, in a mouse model of airway sensitization, RHPS4 treatment attenuates manifestations of allergy decreasing inflammation scores and T cell infiltration in lungs [38]. Since G4 structures are abundant in immunoglobulin switch regions and have a role in recruiting CSR factors and AID, the authors suggest that CSR can be a “druggable” mechanism, and G4 binders may be of interest in immunoallergic diseases.

Recent in vivo studies [112, 113] showed that G4 binders can elicit a downregulation of autophagic pathways in normal post-mitotic neurons, as PDS and Braco-19 (Table 2) decrease the levels of ATG7 mRNA and protein by targeting G4s of the ATG7 gene locus. In addition, G4 binder treatments led to memory loss or defects, and to accelerated ageing of treated mice [112, 113]. The study describes neuronal effects of PDS and Braco-19 investigating a single gene (ATG7), thus it cannot be ruled out that G4 binder effects on neuronal functions are caused by stabilization of DNA/RNA G4s in other genomic loci. Interestingly, the two studied G4 binders showed a different strength in autophagy downregulation [113], suggesting a role for ligand-specific patterns of stabilized G4s or activation of different ligand-specific processes.

Human embryonic stem cells (hESC) show higher G4 levels than differentiated cells, and their homeostatic maintenance may be an important chromatin feature for transcriptional control and cell fate specification [36]. Notably, the authors found that G4 stabilization mediated by PhenDC3 at not cytotoxic concentration causes delayed differentiation of hESC due to failure of pluripotency exit [36]. The results thus emphasize a role of G4 structures in epigenetic regulation of gene expression and cellular differentiation.

Interestingly, these data on G4 binder effects in normal cells have been observed at non-cytotoxic dosage. The low dosage may explain why, despite broader effects could be expected in vivo due to a wide occurrence of G4 structures in the genome, G4 binders appear to have a main specific biological outcome in mice [38, 113]. To discover an effective anticancer immune-stimulation activity of G4 binders, the ligand immunomodulatory effects must be understood in normal cells in which they can instead cause an immune suppressive response, considering in particular the RHSP4 study in B-cell [38]. As PDS stimulates higher levels of IFN-B in breast cancer cells than in lung fibroblasts [35], it will also be interesting to establish whether or not immune gene activation promoted by G4 binders is cancer-cell type specific. In addition, as G4 binders have different effects on autophagy [113], future studies need to define whether those differences are due to ligand structures or targeted G4s.

### Non-canonical DNA structures and topoisomerase-mediated genome instability

Immuno-modulation activity has been proposed also for Topoisomerase poisons arguing that immune-stimulation can contribute significantly to the therapeutic activity of established anticancer agents in patients [114, 115]. In particular, interference of Topoisomerase I (Top1) activity with poisons (camptothecin, CPT) is well known to trigger replication stress, DNA damage and genome instability in an R-loop dependent manner [116–118]. Interestingly, Top1 has been shown to interact with G4 structures [119, 120] and to affect R-loop levels in cells [121, 122]. The mechanism involved in transcription-dependent genome instability has been studied using the murine immunoglobulin switch sequence (Sμ) in yeast [123]. Top 1 deletion resulted into chromosomal aberrations of Sμ regions through R-loop accumulation [123]. As Top 1 can directly bind G4 structures in vitro [119, 120], it may have an important role in controlling R-loop and its cellular depletion results into a G1/S transition block [122, 124]. It is conceivable a molecular mechanism through which a G4/R-loop interplay (forming a G-loop structure) can be functional during transcription to recruit specific enzymes favouring or preventing aberrant genetic rearrangements [22]. Therefore, alterations of homeostatic G-loop dynamics may also be caused by impairment of Top1 interactions with G4 functions [119, 120].

Unbalanced levels of R-loops cause RNA polymerase pausing impairing gene transcription [22, 125]. Interestingly, unscheduled R-loop can be processed by transcription-coupled nucleotide excision repair (TC-NER) endonucleases XPF and XPG [126, 127]. The G4 and R-loop interplay might have a function in driving the DNA damage response as suggested by the fact that G4 binders activity is enhanced in BRCA2-deficient cells [20] and that BRCA2 has been shown to prevent R-loop accumulation [128, 129]. In particular, BRCA2 cooperates with RNA polymerase II during transcription elongation by recruiting PAF1 to the promoter proximal pause sites [129]. Moreover, BRCA2 gene inactivation can cause site-specific formation of unscheduled R-loops and a ERCC4 endonuclease-mediated DNA breaks [129]. On the other hand, BRCA2 represses R-loop mediated replication stress by protecting replication fork from MRE11 degradation [86, 130]. As BRCA2 depletion results into R-loop accumulation, the observation may explain the enhanced G4 binders activity in BRCA2-deficient tumours [20, 25].

Recently, we have shown that low doses of CPT, similarly to G4 binders, induce an increase of micronuclei formation in cancer cells, and the effect is strongly mediated by R-loop formation and DSB [118]. CPT-induced micronuclei lead to the cGAS/STING pathway activation at later times following cell recovery in drug-free medium, then increasing the expression of innate immune genes [118]. Top1 poisons can trigger a higher expression of IRF-3-dependent as well as NF-kB-dependent immune genes, suggesting a complex immune-related effects of Top1 poisons [118]. Gene expression activation is present only in cancer cell lines expressing STING (i.e., HeLa cells), while cells with a marked reduction of STING are resistant to Top1 poison induction of immune genes [118]. Consistently, other published data have shown stimulation of anti-viral immune pathways by Top1 poisons [131]. As STING gene promoter is often methylated in tumours, demethylating agents may increase STING expression and innate immune response upon induction of DNA damage by anticancer agents [132]. Combination of Top1 poisons with immunotherapies is further supported by evidence that in breast cancer cells Topotecan induces IFN I signalling and upregulation of class I MHC genes [133], a potential mechanism for immune sensitization of tumours deficient of MHC antigens on membrane surface. In a set of melanoma models, Top1 poisons have been shown to increase T-cell-mediated cytotoxicity in cells expressing TP53 [134]. In Small Cell Lung Cancer (SCLC) cell lines, only cells expressing STING and cGAS respond to a combination of Top1 poisons and PARP inhibitors with increased mRNA levels of Interferon-B gene [135]. Interestingly, Topotecan, a clinically used Top1 poison, can activate dendritic cells of tumour-bearing mice as exosomes released by the treated tumours are incorporated by dendritic cells, which then triggers the cGAS/STING signalling pathway [136]. Thus, overall the findings suggest that Top1 poisons, at low dosage, may have an immune-stimulatory effect contributing to its established antitumour activity in cancer patients.

### Deregulation of the cGAS-STING pathway in human cancers

Published data support that activation of innate immune response in cancer cells may be exploited to potentiate immunotherapeutic combinations with established antitumour drugs or newly discovered agents, such as G4 binders. However, STING pathways are often impaired in human tumours. STING expression is very often reduced in human SCLC datasets, likely due to promoter methylation, and restoring STING expression with an exogenous gene in SCLC cells did not rescue an innate immune gene response to Top1 poisons, indicating that the STING pathway can be impaired through several mechanisms in SCLC [118]. In addition, it has to be considered that high expression levels of STING can exhibit a strong NF-kB signature that can drive tumorigenesis through chronic inflammation [137]. In particular, genomic DNA can activate a pro-inflammatory pathway promoting a senescence-associated secretory phenotype (SASP) with a long-term genome instability and tissue damage [137–139]. In addition, STING is also involved in non-canonical cGAS-independent mechanisms [140]. It has been demonstrated by interactome analysis with mass spectrometry of breast cancer cells that a part of the cellular STING pool intrinsically resides in the nucleus, prevalently at the inner nuclear membrane, and interacts with the three core proteins of the DNA-PK complex (DNA-PKcs, XRCC6 and XRCC5), a master regulator of DNA damage response [141]. STING may therefore affect genome stability, regulating the DNA damage response by favouring the formation of the non-homologous end joining (NHEJ)-initiation complex at DNA damage sites, promoting breast cancer cell survival and resistance to genotoxic stress [141]. The role of STING in cancer progression and treatment is therefore more complex as it exhibits both tumour-suppressive and tumour-promoting effects [142, 143]. Overall published data show that, in addition to short-term advantages in promoting immune-surveillance, long-term effects of STING pathways can promote cancer progression through chronic inflammation and enhanced immune-suppressive microenvironment. Thus, further investigations will define sub-sets of cancer patients that can likely benefit from immunotherapeutic combinations including effective non-cytotoxic G4 binders as immune-stimulating agents [33].

## Conclusions

**Fig. 4:**
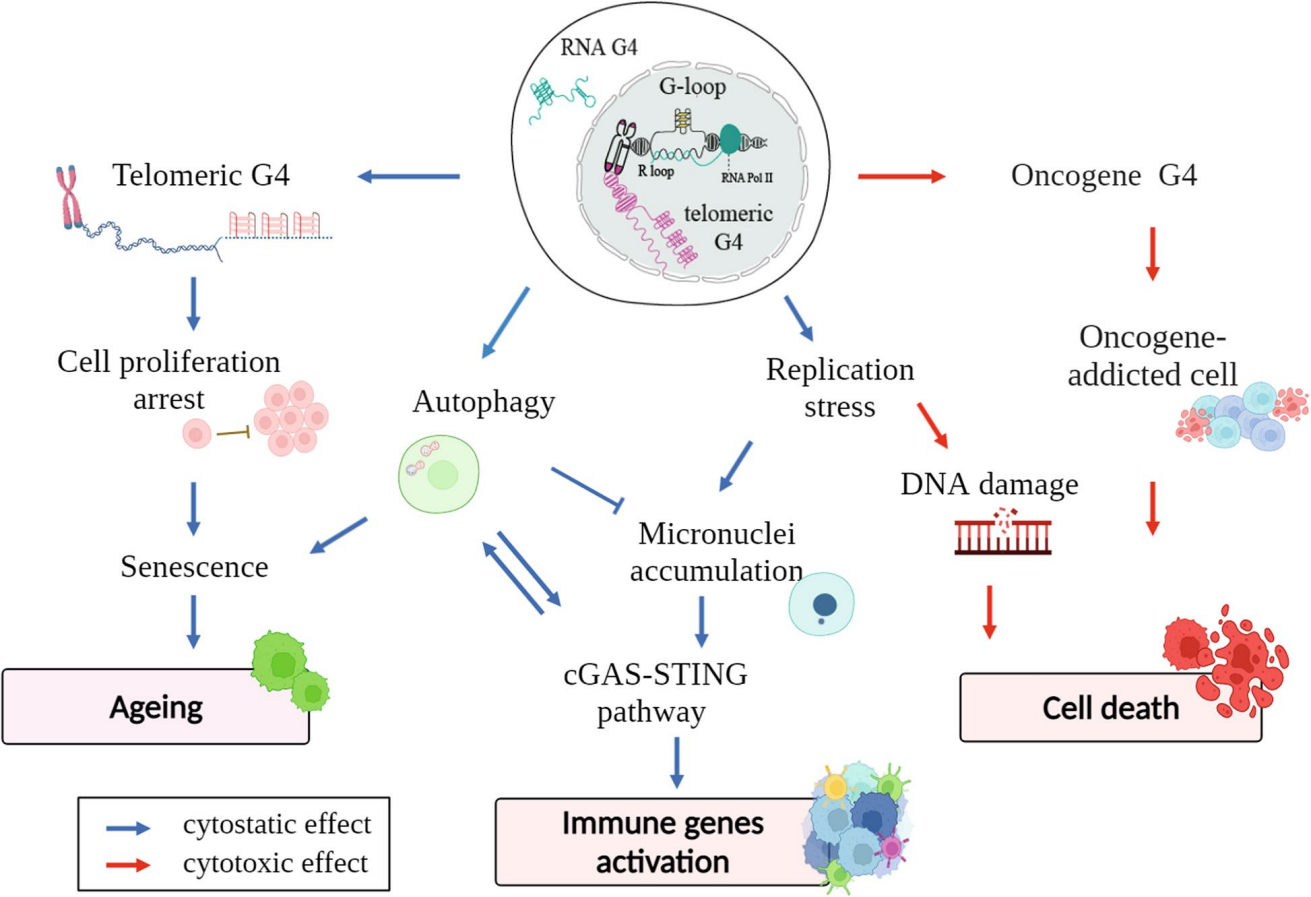
G4 binder-mediated cellular effects. G4-binder mechanisms of action and their pleiotropic cellular outcome as discussed in the review. Created with BioRender.com

G4 structures can form in the genome and transcriptome of normal and cancer cells and are involved in the regulation of several cellular functions. G4s were considered a promising pharmacological target in cancer therapy as they may specifically affect telomere maintenance or expression levels of several oncogenes. However, more recent studies have revealed that G4 binders have pleiotropic effects (Fig. 4). The stabilization of G-loop structures can be a key step of mechanisms leading not only to DNA damage and genome instability, but also to gene expression alterations and telomere interference (Fig. 4). Other mechanisms have also been proposed independently from R loops [22]. In this review, we have discussed how G4 binders could act as enhancers of immune gene expression by harnessing G4-mediated replication and transcription stress in cancer cells at low dosage. We have highlighted how G4 binders may be studied not only for their cell killing activity but instead at their non-cytotoxic doses to optimize immunotherapy efficacy in unresponsive tumours. We therefore propose that G4 binders at low dosage may be used to boost antitumor immunity in patients and may be combined with current standard chemotherapeutics as well as immunotherapeutic strategies. Mutational status and/or expression levels of HR repair genes and cytoplasmic STING pathway genes can be used to select patients more likely to respond to the combination treatment. In addition, low dosages of G4 binders can markedly decrease toxicity due to off-targets. On another side, many questions concerning G4-binder mechanisms of action are still open, thus a full understanding of G4-mediated genome instability and signalling pathways activating innate immune genes will open new horizons for the discovery of effective antitumour G4 binders for a personalized therapy.

## Data Availability

Not applicable.

## Abbreviations

G4: G-quadruplexes
PDS: Pyridostatin
PrimPol: Primase-polymerase
DSB: Double-strand break
HRR: Homologous recombination repair
AGS: Aicardi-Goutières syndrome
AID: Activation-induced deaminase
CPT: Camptothecin
Top1: Topoisomerase 1
SCLC: Small cell lung cancer
